# The RAGE Pathway in Skin Pathology Development: A Comprehensive Review of Its Role and Therapeutic Potential

**DOI:** 10.3390/ijms252413570

**Published:** 2024-12-18

**Authors:** Marcin Radziszewski, Ryszard Galus, Krzysztof Łuszczyński, Sebastian Winiarski, Dariusz Wąsowski, Jacek Malejczyk, Paweł Włodarski, Aneta Ścieżyńska

**Affiliations:** 1 Department of Histology and Embryology, Medical University of Warsaw, 02-004 Warsaw, Poland; 2 Department of Thoracic Surgery, National Medical Institute of the Ministry of the Interior and Administration, 02-507 Warsaw, Poland; 3 Laboratory of Molecular Oncology and Innovative Therapies, Military Institute of Medicine National Research Institute, 04-141 Warsaw, Poland; 4 Institute of Health Sciences, Faculty of Medical and Health Sciences, University of Siedlce, 08-110 Siedlce, Poland

**Keywords:** RAGE, skin diseases, fibrosis, inflammation, skin neoplasms

## Abstract

The receptor for advanced glycation end-products (RAGE), a member of the immunoglobulin superfamily, is expressed in various cell types and mediates cellular responses to a wide range of ligands. The activation of RAGE triggers complex signaling pathways that drive inflammatory, oxidative, and proliferative responses, which are increasingly implicated in the pathogenesis of skin diseases. Despite its well-established roles in conditions such as diabetes, cancer, and chronic inflammation, the contribution of RAGE to skin pathologies remains underexplored. This review synthesizes current findings on RAGE’s involvement in the pathophysiology of skin diseases, including conditions such as psoriasis, atopic dermatitis, and lichen planus, focusing on its roles in inflammatory signaling, tissue remodeling, and skin cancer progression. Additionally, it examines RAGE-modulating treatments investigated in dermatological contexts, highlighting their potential as therapeutic options. Given RAGE’s significance in a variety of skin conditions, further research into its mediated pathways may uncover new opportunities for targeted interventions in skin-specific RAGE signaling.

## 1. Introduction

The receptor for advanced glycation end-products (RAGE) is a multiligand cell surface receptor that plays a central role in mediating inflammation and immune responses. As part of the immunoglobulin superfamily, RAGE can bind a wide range of molecules, including advanced glycation end-products (AGEs), advanced oxidation protein products (AOPPs), S100/calgranulins, high-mobility group box 1 (HMGB1), and amyloid-beta peptides [1]. Initially recognized for its role in diabetic complications and neurodegenerative diseases [1], growing evidence now suggests that the RAGE pathway is critically involved in the pathogenesis of various skin disorders [2].

The skin, being the largest organ and a key barrier against environmental insults, is constantly exposed to factors that trigger inflammatory responses. Chronic inflammation underpins many skin diseases, including autoimmune conditions such as lupus, psoriasis, and lichen planus, as well as infectious diseases and wound-healing disorders [3]. The RAGE pathway, known for its contribution to sustained inflammation, may serve as a key driver in these processes, influencing both local and systemic immune responses [2].

Despite its established role in other inflammatory and metabolic diseases [4,5], the study of RAGE in dermatological contexts remains in its early stages. Current research, although limited, suggests that RAGE and its related soluble forms may contribute to the onset and progression of various skin conditions, including inflammatory and autoimmune disorders, impaired wound healing, and microbial infections. These insights have opened new possibilities for targeting the RAGE pathway as a therapeutic strategy in the management of skin diseases.

This review provides a comprehensive overview of the RAGE pathway’s involvement in skin disorders, drawing from a systematic review of the literature (Supplementary Figure S1). By synthesizing current research, we examine the molecular mechanisms by which RAGE contributes to skin pathology, evaluate its potential as a diagnostic marker, and discuss emerging therapeutic prospects. Given the complexity and multifactorial nature of skin diseases, understanding RAGE’s role may offer new insights into disease pathogenesis and pave the way for innovative treatment strategies.

## 2. Receptor and Its Ligands

The RAGE gene encodes a receptor belonging to the immunoglobulin superfamily, primarily recognized for binding advanced glycation end-products (AGEs) [6]. AGEs are heterogeneous molecules naturally formed throughout human life via a nonenzymatic reaction involving proteins, sugars, lipids, and nucleic acids [7]. However, this process can be accelerated by factors such as oxidative stress and hyperglycemia, commonly observed in several chronic diseases. RAGE is classified as a type I transmembrane glycoprotein that transduces extracellular signals into the cell, leading to the activation of pro-inflammatory gene expression [8].

**Figure 1:**
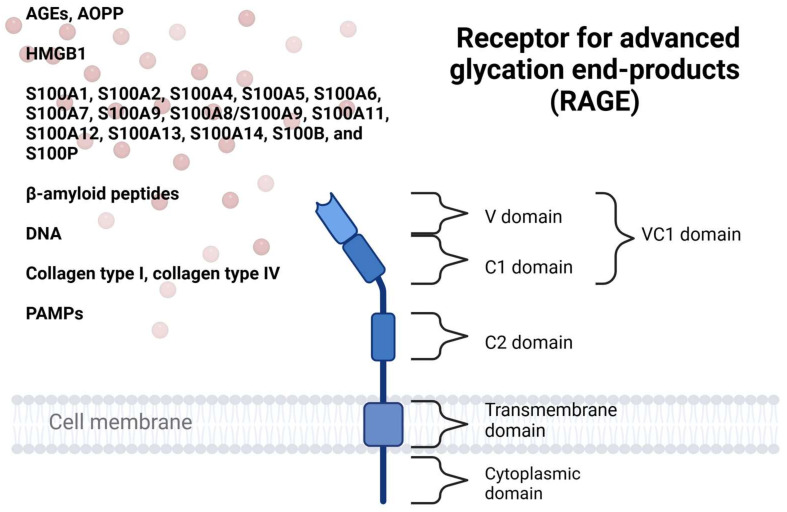
The cell membrane receptor for advanced glycation end-products (RAGE) consists of a VC1 domain, a C2 domain, and both transmembrane and intracellular regions. RAGE binds a range of endogenous danger-associated molecular patterns (DAMPs) and exogenous pathogen-associated molecular patterns (PAMPs) through its positively charged VC1 domain. Key ligands include advanced glycation end-products (AGEs), advanced oxidation protein products (AOPPs), high-mobility group box 1 (HMGB1), S100 family proteins, beta-amyloid proteins, DNA, and collagen. Created with BioRender.com (accessed on 14 November 2024).

In addition to binding various AGEs, RAGE functions as a pattern recognition receptor, analogous to Toll-like receptors (TLRs) [9]. It responds to a wide range of danger-associated molecular patterns (DAMPs) from damaged host cells and pathogen-associated molecular patterns (PAMPs) from infectious microorganisms [10]. Upon ligand binding, RAGE primarily initiates inflammatory cellular activation through the NF-κB transcription factor. Furthermore, it facilitates the recruitment of inflammatory cells by acting as an adhesive receptor, interacting with leukocyte β2-integrins [11]. The structure of the RAGE receptor and its primary ligands are depicted in Figure 1.

RAGE ligands can be broadly categorized as exogenous or endogenous. Exogenous ligands include a heterogeneous group of molecules derived from pathogens and foreign substances. In contrast, endogenous ligands are produced within the body and include AGEs, high-mobility group box 1 protein (HMGB1), members of the S100 protein family, amyloid β peptide, and type I and type IV collagens [12].

HMGB1, also known as amphoterin or nerve axon growth factor, is a DNA-binding protein released by damaged and dying cells, functioning as a DAMP [13]. It binds to RAGE and TLRs on the surface of various immune cells, triggering the release of pro-inflammatory cytokines [14]. In addition to being passively released by dying cells, HMGB1 can be actively secreted by macrophages, dendritic cells, natural killer cells, and neutrophils in response to stimuli such as tumor necrosis factor α (TNFα), interleukin-1 (IL-1), and endotoxins [15,16]. Beyond its role in inflammation, HMGB1 also acts as a transcription factor and contributes to chromatin remodeling [17].

The S100 family, also known as calgranulins, comprises calcium-binding proteins released by various cell types, playing roles in differentiation, proliferation, migration, and inflammation [18]. To date, 25 S100 proteins have been identified, though many remain incompletely characterized [18]. Several family members, including S100A1, S100A2, S100A4 (Metastasin), S100A5, S100A6, S100A7 (psoriasin), S100A8, S100A9, S100A8/S100A9 (Calprotectin), S100A11, S100A12, S100A13, S100A14, S100B, and S100P, are recognized as ligands for RAGE [19]. Notably, S100A7 (psoriasin) is overexpressed in hyperproliferative skin disorders, modulating cytokine and chemokine production [20]. In contrast, S100A4 (Metastasin) and S100A13 are implicated in promoting tumor cell survival and metastasis in cancers, including melanoma [19,21]. Additionally, S100A12, also known as extracellular newly identified RAGE-binding protein (EN-RAGE), may serve as an inflammatory marker [22].

Other RAGE ligands include β-amyloid peptides, which play a critical role in the pathogenesis of Alzheimer’s disease [23,24], and type I and type IV collagens, which contribute to the dispersion of lung angiotensin type 1 stem cells [25]. RAGE also binds extracellular DNA, acting as a DAMP, with its activation being even more potent when complexed with HMGB1 [26]. Furthermore, RAGE interacts with various foreign molecules classified as PAMPs, emphasizing its role in the innate immune response, akin to that of TLRs [14].

## 3. Molecular Pathway and Isoforms of RAGE

**Figure 2:**
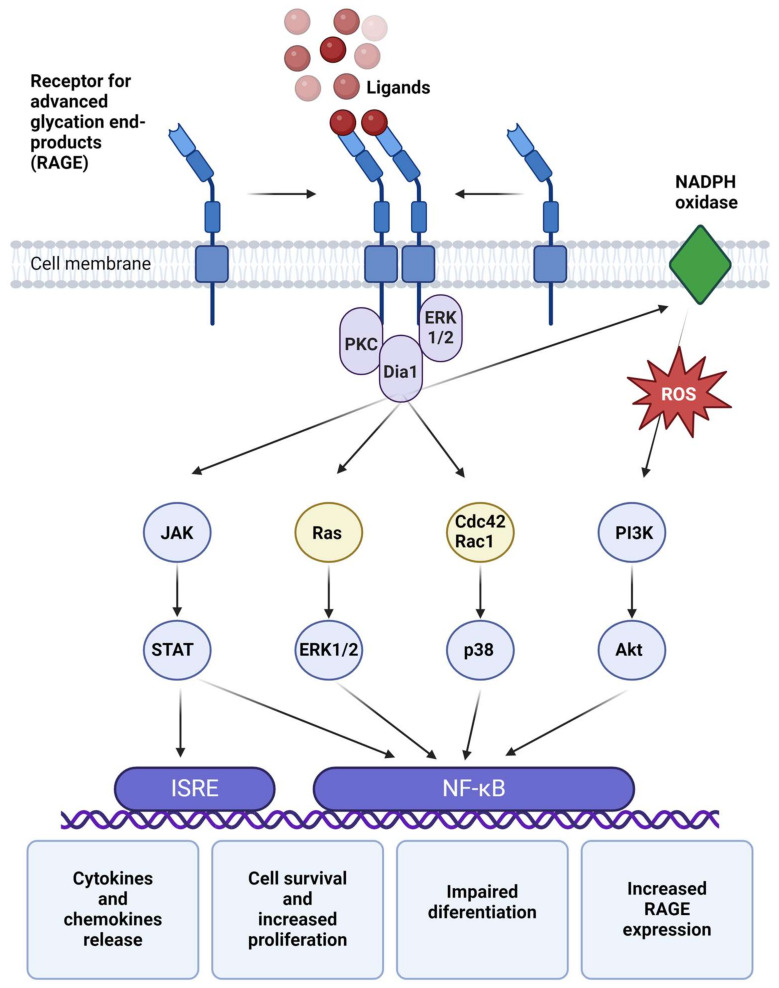
RAGE forms oligomers to bind ligands, interacting with negatively charged molecules. Upon ligand binding, conformational changes occur in the receptor’s cytoplasmic tail, activating signaling adaptors such as diaphanous-1 (Dia1), extracellular signal-regulated kinase (ERK1/2), and protein kinase C (PKC). Dia1 subsequently activates small GTPases like Ras, Cdc42, and Rac1, which stimulate NF-κB signaling via the ERK1/2 and p38 MAPK pathways. NF-κB activation may also occur through the PI3K/Akt pathway, often triggered by reactive oxygen species (ROS) generated during RAGE signaling. Additionally, Dia1 can engage the JAK/STAT pathway, activating both NF-κB and interferon-stimulated response elements (ISRE), amplifying the inflammatory response. These cascades result in pro-inflammatory cellular changes and chemotaxis, recruiting additional inflammatory cells. Created with BioRender.com (accessed on 14 November 2024).

It is believed that the membrane-bound form of the RAGE receptor must form oligomers to activate signaling pathways. Ligand binding primarily occurs through electrostatic interactions between the receptor’s positively charged surface and negatively charged ligands [27]. Upon binding, alterations in the receptor’s intracellular domain affect various adapter molecules, including Dia1, ERK1/2, and PKC [27,28]. Dia1 subsequently activates Rho family GTPases, such as Cdc42 and Rac1, which stimulate the NF-κB transcription factor via p38 kinase [28]. Additionally, RAGE activates NF-κB through the classical mitogen-activated protein kinase (MAPK) pathway involving Ras GTPase and ERK1/2 [12]. NF-κB activation can also occur via NADPH oxidase, which activates the PI3K and Akt pathways [27]. Furthermore, RAGE stimulation triggers the JAK/STAT pathway, which not only enhances NF-κB activity but also activates interferon-stimulated response elements (ISRE), further amplifying inflammation [29]. Notably, NF-κB induces RAGE receptor expression, creating a feedback loop that exacerbates the inflammatory response [29]. The gene expression profile activated by RAGE promotes the release of cytokines and chemokines, enhances cell survival and proliferation, and impairs differentiation [1]. Figure 2 outlines the intracellular processes triggered by RAGE activation.

In contrast to full-length RAGE (fl-RAGE), the soluble form of RAGE (sRAGE) lacks the transmembrane domain, allowing it to circulate freely in the bloodstream. Unlike membrane-bound RAGE, which facilitates direct cell signaling, sRAGE acts as a decoy receptor by binding RAGE ligands and preventing their interaction with membrane-bound RAGE, thereby inhibiting downstream signaling pathways [30]. A similar role is attributed to endogenous secretory RAGE (esRAGE), a soluble isoform actively secreted by cells and produced through the alternative splicing of the RAGE gene. Both sRAGE and esRAGE mitigate the harmful effects of RAGE activation by sequestering ligands, reducing inflammation and cellular damage in diseases such as diabetes, cardiovascular disorders, and neurodegenerative conditions [31]. Elevated levels of sRAGE and esRAGE have been associated with these diseases, positioning them as potential biomarkers for disease progression [32,33]. While soluble RAGE forms possess protective properties, elevated levels may reflect disease severity, potentially serving as a compensatory mechanism to counteract further progression [34].

Another RAGE isoform is dominant-negative RAGE (dnRAGE), a membrane-bound receptor variant that lacks the cytoplasmic tail necessary for signal transduction. Although dnRAGE cannot participate in signaling, it retains its adhesive properties. Recent studies suggest that a shift from fl-RAGE to dnRAGE plays a role in metastasis and tumor progression. In lung adenocarcinoma, this shift is characterized by reduced fl-RAGE levels and elevated dnRAGE levels, accompanied by upregulated vimentin and decreased expression of proteins involved in cell polarization—factors that promote increased cell motility and invasiveness [35]. However, the mechanism by which dnRAGE inhibits or reverses fl-RAGE expression remains poorly understood. Additionally, dnRAGE may function as a decoy receptor, sequestering pro-inflammatory RAGE ligands and modulating inflammatory responses [11].

Additional RAGE isoforms can arise through alternative splicing or proteolytic processing, though these are less well characterized and typically represent variations of the primary forms [36]. Techniques such as quantitative reverse transcription PCR (qRT-PCR), using primers designed for unique transcript regions, and Western blotting, employing antibodies targeting specific receptor epitopes—such as the N-terminal, C-terminal, or transmembrane domains—are effective for differentiating between these isoforms [37,38].

## 4. RAGE Involvement in Skin Pathologies

Although RAGE is present in healthy human skin, its expression levels correlate with skin thickness and the concentrations of inflammatory markers such as TNFα, IL-1, and S100B [39]. In rodent models, the specific deletion of RAGE in keratinocytes has been shown to reduce TNFα expression and accelerate the resolution of inflammation [40]. Similarly, in HaCaT cells, exposure to AGEs and UVB light induces inflammaging, a chronic, low-grade inflammatory state associated with aging, characterized by increased RAGE expression and elevated levels of pro-inflammatory cytokines, including IL-1β, IL-6, and TNFα [41].

Additionally, in inflamed skin, RAGE ligands, such as S100A7, are overexpressed in keratinocytes, as well as in dendritic and stromal cells within the dermis [42]. Recent studies by Han et al. demonstrated that echinacoside–zinc coordination polymers can reduce RAGE expression and mitigate skin inflammation [43]. Small-molecule drugs targeting RAGE ligands, such as HMGB1, are also proposed to reduce cutaneous inflammation [44].

Overall, RAGE plays a well-recognized role in chronic inflammation, with increasing evidence supporting its involvement in localized inflammatory processes within the skin, underscoring its potential as a therapeutic target in various dermatological conditions.

### 4.1. Psoriasis

Psoriasis is a chronic autoimmune skin disorder characterized by the accelerated proliferation of keratinocytes, resulting in the formation of thick, erythematous, scaly plaques that are associated with inflammation and can affect various areas of the body. While the involvement of the RAGE pathway in psoriasis is not yet fully understood, the existing literature suggests that it plays a significant role in the disease’s pathogenesis. A recent study by Kang et al. demonstrated elevated levels of AGEs in both the serum and psoriatic plaques, highlighting that AGE accumulation in plaques stimulates IL-36α production in keratinocytes, which may subsequently activate Th17 cells—key drivers of psoriasis pathogenesis [45].

Similarly, Papagrigoraki et al. observed higher concentrations of AGEs in both the serum and psoriatic plaques of patients, suggesting that the AGE–RAGE axis may play a key role in the pathogenesis of psoriasis. Furthermore, they linked the release of AGEs from the skin to the elevated metabolic and cardiovascular risks observed in psoriatic patients [46]. The activation of the AGE–RAGE axis triggers the release of reactive oxygen species, leading to endothelial dysfunction, persistent inflammation in the coronary arteries and heart tissue, and direct myocardial damage [46]. Additionally, severe psoriasis was found to be inversely correlated with serum sRAGE concentration, likely due to an increased concentration of RAGE ligands that block the ligand-binding domain of RAGE, thereby reducing its availability [46].

Conversely, other studies analyzing the serum of psoriatic patients have reported not only increased levels of AGEs but also elevated sRAGE levels [47,48]. These findings underscore the complex and not fully understood role of sRAGE and esRAGE, which can simultaneously mitigate the disease by sequestering ligands while also serving as markers of disease severity. Further research is needed to delineate the distinct roles of RAGE isoforms in psoriasis. Table 1 provides an overview of current data on RAGE involvement in psoriasis from human-based studies, emphasizing its potential role in the disease’s pathogenesis. Interestingly, the expression of fl-RAGE in psoriatic plaques remains inconclusive, as studies have yet to provide definitive evidence.

Moreover, Strohbuecker et al. found that skin biopsies from psoriatic patients display elevated levels of the RAGE ligand HMGB1, alongside increases in IL-17 and CD3-positive cells, both of which are recognized as significant contributors to psoriasis development [49]. Additionally, peripheral blood samples from psoriatic patients revealed enhanced RAGE expression in CD4 and CD8-positive leukocytes [49], with the 2184G allele of the 2184A/G RAGE polymorphism identified as a significant psoriasis risk factor [50].

Several RAGE-binding calgranulins, including S100B, S100A2, S100A4, S100A7, S100A8, S100A9, and S100A12, have been found to be elevated in both the serum and skin biopsies of psoriatic patients [51,52,53,54,55,56]. Among these, S100A7 (psoriasin) is particularly noteworthy for its role in psoriasis. S100A7 not only inhibits keratinocyte differentiation but also promotes IL-6 release through NF-κB activation and stimulates IL-1 release via the p38 MAPK pathway [57,58]. Wolf et al. discovered that genetically modified mice with the epidermal overexpression of S100A7 exhibited heightened inflammatory responses to abrasion, resembling the cellular and cytokine profiles observed in psoriasis [42].

Despite the elevated concentration of S100A7 in psoriatic lesions, serum levels of this protein may decline as the disease progresses. This reduction could be attributed to an increase in anti-psoriasin antibodies, which may influence the overall dynamics of S100A7 within the body [59]. This observation underscores the complex interplay between local inflammation and systemic immune responses in psoriasis.

**Table 1:** Summary of current human-based studies on RAGE involvement in psoriasis, emphasizing its potential role in disease pathogenesis (↑—increased concentration, ↓—decreased concentration).

Material	Method	Study Size	Markers	Year	Authors [Ref.]
Serum	ELISA	60	↑AGEs	2023	Kang et al. [45]
Serum	ELISA	75	↑CML, ↑CEL, ↑sRAGE	2022	Damasiewicz-Bodzek et al. [47]
Serum	ELISA	160	↑AGEs, ↑sRAGE	2022	Karas et al. [48]
Serum	ELISA	158	↑HMGB1, ↑S100A7, ↑S100A12	2020	Borsky et al. [54]
Serum	ELISA	80	↑AGEs, ↓sRAGE, ↓esRAGE	2017	Papagrigoraki et al. [46]
Serum	ELISA	50	↑S100B	2017	Salem et al. [51]
Serum	ELISA	55	↑S100A7, ↑S100A8/A9, ↑S100A12	2016	Wilsmann-Theis et al. [55]
Serum	ELISA	14	↑S100A8/S100A9, ↓S10A7	2009	Anderson et al. [59]
Peripheral leukocytes	Flow cytometry	32	↑RAGE	2019	Strohbuecker et al. [49]
Peripheral leukocytes	PCR	272	↑2184G RAGE allele	2002	Vasku et al. [50]
Skin biopsy	IHC	10	↑AGEs	2023	Kang et al. [45]
Skin biopsy	IHC	14	↑HMGB1	2019	Strohbuecker et al. [49]
Skin biopsy	IHC	80	↑AGEs	2017	Papagrigoraki et al. [46]
Skin biopsy	IHC	50	↑S100B	2017	Salem et al. [51]
Skin biopsy	PCR	341	↑S100A7, ↑S100A8, ↑S100A9, ↑S100A12	2016	Wilsmann-Theis et al. [55]
Skin biopsy	Western Blot, PCR	21	↑S100A4	2010	Zibert et al. [53]
Skin biopsy	IHC	14	↑S100A7	2009	Anderson et al. [59]

Abbreviations: advanced glycation end-products (AGEs), Enzyme-Linked Immunosorbent Assay (ELISA), High Mobility Group Box 1 (HMGB1), immunohistochemistry (IHC), N6-Carboxyethyllysine (CEL), N6-Carboxymethyllysine (CML), Polymerase Chain Reaction (PCR), receptor for advanced glycation end-products (RAGE), soluble RAGE (sRAGE), endogenous secretory RAGE (esRAGE).

### 4.2. Atopic Dermatitis

Atopic dermatitis (AD) is a chronic inflammatory skin condition characterized by itchy, red, dry patches, often triggered by environmental factors and associated with a heightened immune response, commonly seen in individuals with a personal or family history of allergies. The literature on the RAGE pathway in AD is limited, with only a few human-based studies available.

Research by Hong et al. demonstrated that AD patients exhibit significantly elevated concentrations of AGEs in corneocytes from both lesional and non-lesional tissue; however, serum levels of AGEs were comparable to those in healthy controls [60]. In contrast to psoriasis, serum concentrations of sRAGE were found to be significantly lower in AD patients, a finding supported by subsequent studies [60,61]. Moreover, reduced sRAGE levels have been associated with the progression of urticarial conditions [62]. Table 2 summarizes the current data on the involvement of RAGE in AD based on human studies, emphasizing its potential role in the disease’s pathogenesis.

Additionally, Jin et al. demonstrated that S100A9 activates keratinocytes via the RAGE receptor, inducing IL-33 secretion and amplifying the Th2 immune response characteristic of AD. Their study also reported significantly elevated levels of S100A9 and the S100A8/A9 complex in both the lesional skin and serum of AD patients compared to healthy controls, whereas S100A8 levels alone remained relatively unchanged [63]. Similarly, increased levels of other calcium-binding proteins, such as S100A2 and S100A12, have been observed in AD [52,64].

Notably, S100A7 levels are elevated in lesional AD tissue, with immunostaining intensities comparable to those seen in psoriasis [65]. These findings underscore a complex interplay between RAGE ligands and immune responses in AD, highlighting the need for further research into their potential as therapeutic targets.

**Table 2:** Summary of current human-based studies on RAGE involvement in atopic dermatitis (AD), emphasizing its potential role in disease pathogenesis (↑—increased concentration, ↓—decreased concentration).

Material	Method	Study Size	Markers	Year	Authors [Ref.]
Serum	ELISA	65	↓sRAGE	2023	Eke-Gungor et al. [61]
Serum	ELISA	41	↓sRAGE, ↓esRAGE	2020	Hong et al. [60]
Serum	ELISA	15	↑S100A9, ↑S100A8/A9	2014	Jin et al. [63]
Lesion washing fluids	ELISA	12	↑S100A7	2009	Gläser et al. [65]
Skin biopsy	ELISA	41	↑AGEs	2020	Hong et al. [60]
Skin biopsy	IHC	15	↑S100A9, ↑S100A8/A9	2014	Jin et al. [63]
Skin biopsy	IHC, PCR	51	↑S100A12	2013	Suárez-Fariñas et al. [64]
Skin biopsy	IHC	4	↑S100A7	2009	Gläser et al. [65]

Abbreviations: advanced glycation end-products (AGEs), Enzyme-Linked Immunosorbent Assay (ELISA), endogenous secretory RAGE (esRAGE), immunohistochemistry (IHC), Polymerase Chain Reaction (PCR), receptor for advanced glycation end-products (RAGE), soluble RAGE (sRAGE).

### 4.3. Lichen Planus

Lichen planus (LP) is a chronic inflammatory autoimmune disease characterized by flat, purple, pruritic plaques that can affect the skin, mucous membranes, and nails [66]. Although its pathogenesis remains unclear, LP has been associated with certain viral infections, including human herpesvirus type 7 (HHV-7) and hepatitis C virus (HCV) [67,68]. Additionally, various alarmins are suspected to contribute to disease development by promoting the recruitment of CD8+ T cells through pattern recognition receptors such as TLRs and RAGE, resulting in inflammatory infiltration within lesions [69]. Table 3 summarizes recent human studies examining the role of RAGE in LP and its potential contribution to disease pathogenesis. Notably, RAGE expression has only been assessed using immunohistochemistry (IHC), and it remains unclear whether the isoform detected was fl-RAGE.

Recently, HMGB1 has emerged as a key DAMP involved in LP pathogenesis. In a study by De Carvalho et al., immunohistochemical analysis revealed elevated levels of HMGB1, TLR4, and RAGE in the dermis of cutaneous LP patients compared to healthy controls, while epidermal concentrations of these molecules were comparable between the groups [70]. However, serum HMGB1 levels were not elevated in the LP group, suggesting its involvement is primarily locoregional [70].

Similarly, in the mucosal form of the disease, histological examination revealed an increased expression of HMGB1, RAGE, and TLR4. These molecules were also elevated within the epithelial layers, with the most significant increases observed in the subepithelial inflammatory infiltrate [71].

### 4.4. Cholesteatoma

In cholesteatoma, abnormal growth of keratinizing squamous epithelium occurs within the middle ear, leading to chronic inflammation, debris accumulation, and recurrent infections [72]. Szczepański et al. conducted a comparative study involving cholesteatoma samples and skin specimens from the external auditory canal and retroauricular area. Their findings revealed a significantly elevated expression of RAGE and HMGB1 in cholesteatoma epithelium compared to normal skin [73]. Immunostaining demonstrated that RAGE expression in normal skin was either absent or weak, limited to the basal and granular layers and sebaceous glands. In contrast, all epithelial layers of the cholesteatoma samples exhibited RAGE positivity. The authors proposed that HMGB1 and RAGE overexpression in cholesteatoma promotes increased keratinocyte proliferation, survival, and migration via the activation of the PI3K/Akt/NF-κB, MAPK p44/p42, Erk1/2, and STAT3 signaling pathways, while also enhancing IL-8 release [73].

Further research supports the involvement of the RAGE pathway in cholesteatoma development. A study comparing plasma-derived small extracellular vesicles (sEVs) from cholesteatoma patients and healthy controls detected higher HMGB1 levels in the sEVs from the patient group [74]. Moreover, sEV-derived HMGB1 was found to more effectively stimulate keratinocyte proliferation and IL-6 production in a cell line model compared to pure HMGB1 particles [74].

Overall, the RAGE pathway appears to play a critical role in cholesteatoma development, although further studies are needed to validate these findings and clarify its precise mechanisms.

### 4.5. Skin Infection

In addition to studies linking the RAGE pathway to skin inflammation, several investigations have examined its role in skin infections. Achouiti et al. suggest that while RAGE plays a limited role in local host defense against skin infections, it may contribute to distant bacterial outgrowth [75]. In a rodent model of Staphylococcus aureus skin infection, RAGE-deficient mice exhibited significantly lower bacterial counts in distant organs, suggesting that RAGE may facilitate bacterial dissemination and growth in systemic infections [75].

**Figure 3:**
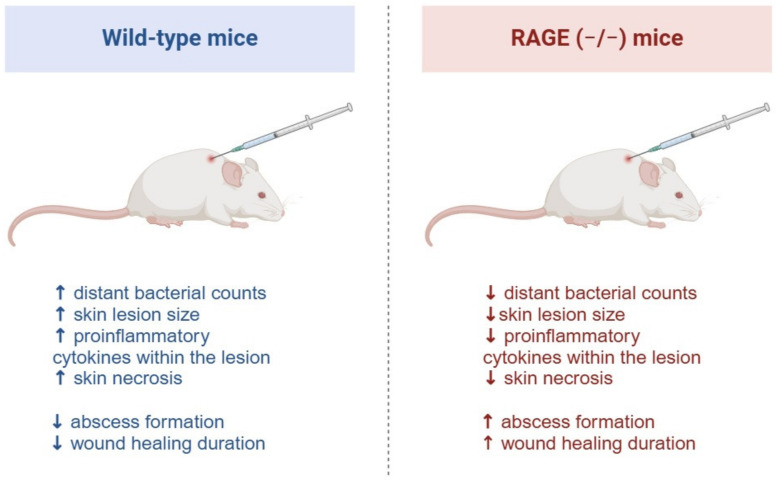
Comparison of RAGE-deficient and wild-type mice responses to subcutaneous *Staphylococcus aureus* injection reveals that RAGE facilitates distant bacterial migration and exacerbates local tissue damage during infection (↑—increase, ↓—decrease). Conversely, RAGE activation in infection-related wounds may support the healing process, indicating a dual role for the receptor depending on context. Created with BioRender.com (accessed on 14 November 2024).

Na et al. reported that RAGE-deficient mice infected with *S. aureus* developed smaller skin lesions and exhibited lower levels of pro-inflammatory cytokines [76]. However, RAGE deficiency also resulted in a higher number of abscesses and prolonged wound healing. The authors proposed that the absence of RAGE enhances the phagocytic activity of phagocytes, thereby reducing bacterial load in the skin and mitigating lesion severity. Figure 3 illustrates the key differences in response to *S. aureus* skin infection between RAGE-deficient and wild-type mice.

Further research into RAGE expression in leprosy patients revealed a predominantly pro-inflammatory role for the receptor, rather than an antimycobacterial effect. An analysis of RAGE and S100A12 expression in the skin and serum of leprosy patients suggested that targeting the RAGE pathway may help to prevent complications and tissue damage associated with the disease [77].

The role of the RAGE pathway in infection appears to vary depending on the site of infection and the type of pathogen involved [78,79,80]. One critical mechanism by which RAGE may influence infection is its ability to bind β2-integrins, promoting neutrophil infiltration into infected tissue [75].

### 4.6. Skin Fibrosis

The primary mechanism driving fibrotic changes across various tissues is the excessive secretion of extracellular matrix (ECM) components by activated myofibroblasts, coupled with their contractile activity. While the TGF-β-dependent SMAD pathway is a key promoter of ECM production and fibroblast survival, growing evidence suggests that the RAGE/NF-κB axis also plays a significant role in fibrosis development through ERK1/2- and MAPK-dependent fibroblast activation and increased TGF-β production [81,82]. The antifibrotic effects of sRAGE further underscore the involvement of the RAGE pathway in this process [83]. Additionally, the activation of the RAGE/NF-κB axis enhances the release of matrix metalloproteinases (MMPs), facilitating tissue remodeling [81,83]. However, in a profibrotic environment, the overall balance shifts toward ECM accumulation. Table 4 summarizes studies demonstrating the involvement of the RAGE pathway in skin fibrotic changes.

Recent studies highlight that the S100A4-dependent activation of RAGE and TLRs plays a critical role in fibrosis development in the lungs, liver, kidneys, and heart, and is also implicated in the progression of systemic sclerosis (SSc) [81]. Tomcik et al. observed that S100A4 induces an active fibroblast phenotype and stimulates the TGF-β/SMAD pathway [84]. Furthermore, their study showed that S100A4 deficiency in mouse models significantly reduces fibrosis, suggesting that S100A4-neutralizing antibodies could serve as a potential therapeutic target. Recent findings confirm that anti-S100A4 monoclonal antibodies can reverse fibrosis by reducing dermal thickness, decreasing myofibroblast counts, and lowering collagen accumulation in murine models of pre-established bleomycin-induced skin fibrosis [85,86].

Similarly, elevated levels of S100A9 have been detected in the skin tissues of mice with scleroderma, with its mechanism of action linked to the RAGE/ERK1/2/NF-κB signaling pathway [87]. In a rabbit model of hypertrophic scars, the pharmacological inhibition of S100A12, RAGE, and TLR4 limited fibroblast activation [88]. In a rodent model, alternatively activated macrophage-derived HMGB1 was shown to stimulate α2-antiplasmin (α2AP) production and fibrosis through the RAGE pathway and IL-4 secretion. Moreover, IL4Rα-neutralizing antibodies attenuated fibrotic changes in bleomycin-induced mice [89].

**Table 4:** Summary of research highlighting the role of the RAGE pathway in skin fibrosis (↑—increased concentration).

Model	Material	Condition	Method	Markers	Year	Authors [Ref.]
Human	Serum	Systemic sclerosis	ELISA, PCR	↑S100A8, ↑S100A9	2013	Xu et al. [90]
Human	Serum	Systemic sclerosis	ELISA	↑HMGB1, ↑sRAGE, ↑IgG, ↑CRP	2009	Yoshizaki et al. [91]
Human	Serum	Systemic sclerosis	ELISA	↑CML	2007	Kaloudi et al. [92]
Human	Skin biopsy	Systemic sclerosis	IHC, PCR, Western Blot	↑S100A4	2015	Tomcik et al. [84]
Human	Skin biopsy	Systemic sclerosis	IHC	↑S100A8, ↑S100A9, ↑RAGE	2013	Xu et al. [90]
Human	Skin biopsy	Systemic sclerosis	IHC	↑HMGB1, ↑RAGE	2009	Yoshizaki et al. [91]
Human	Skin biopsy	Hypertrophic scar and keloid	IHC	↑S100A12	2017	Zhao et al. [88]
Murine	Serum	Bleomycin-induced fibrosis	ELISA	↑HMGB1, ↑sRAGE	2009	Yoshizaki et al. [91]
Murine	Skin biopsy	Bleomycin-induced fibrosis	IHC, Western Blot	↑HMGB1, ↑iNOS, ↑IL-4, ↑Arg-1, ↑α2AP, ↑α-SMA, ↑type I collagen	2020	Kanno et al. [89]
Murine	Skin biopsy	Bleomycin-induced fibrosis	PCR, Western Blot	↑S100A9, ↑IL-6, ↑IL-1β, ↑IL-8, ↑TNF-α	2018	Xu et al. [87]
Murine	Skin biopsy	Bleomycin-induced fibrosis	IHC, PCR, Western Blot	↑S100A4	2015	Tomcik et al. [84]

Abbreviations: Alpha-2-Antiplasmin (α2AP), Alpha-Smooth Muscle Actin (α-SMA), Arginase-1 (Arg-1), C-Reactive Protein (CRP), High Mobility Group Box 1 (HMGB1), fImmunoglobulin G (IgG), immunohistochemistry (IHC), Inducible Nitric Oxide Synthase (iNOS), Interleukin (IL), N6-Carboxymethyllysine (CML), Polymerase Chain Reaction (PCR), Receptor for advanced glycation end-products (RAGE), soluble RAGE (sRAGE).

In humans, the increased expression of S100A12 has been observed in the epidermis of hypertrophic and keloid scars [88]. Xu et al. demonstrated elevated levels of S100A8, S100A9, and RAGE in sclerotic skin through immunohistochemistry. Plasma concentrations of S100A8 and S100A9 were higher in patients with diffuse cutaneous systemic sclerosis (dcSSc) compared to those with limited cutaneous systemic sclerosis (lcSSc) and healthy controls. The transcription levels of these markers were elevated across all SSc cases [90]. Similarly, Yoshizaki et al. showed that the plasma levels of HMGB1 and sRAGE are increased in patients with SSc, with disease severity correlating with these serological changes [91].

Kaloudi et al. further observed higher serum levels of AGEs in SSc patients compared to healthy controls [92]. The deposition of AGEs in the dermis of SSc patients appears to contribute to the pathogenesis of calcinotic deposits, which are believed to exacerbate tissue ischemia and disease progression [93].

Overall, the RAGE pathway plays a significant role in skin fibrotic processes, making it a promising target for the development of future therapeutic interventions.

### 4.7. Diabetic Wound Formation and Healing

**Figure 4:**
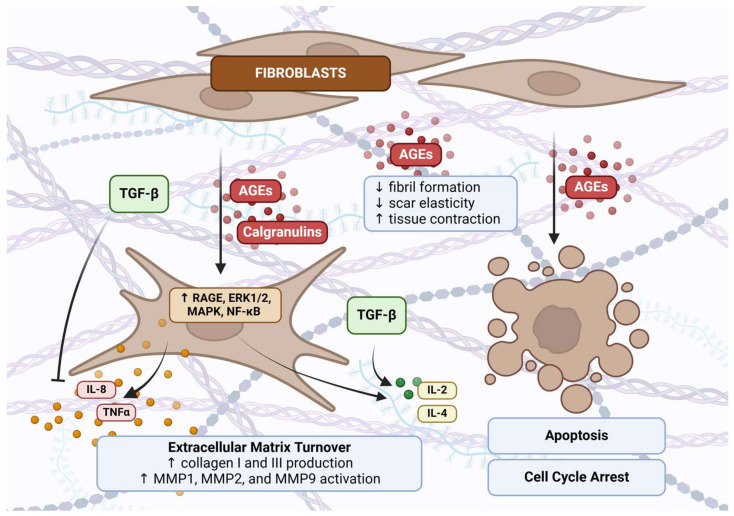
Overview of RAGE pathway involvement in wound healing (↑—increase, ↓—decrease). The excessive accumulation of AGEs in the skin disrupts fibril formation and scar elasticity, leading to increased tissue contraction. AGEs induce fibroblast apoptosis and cause cell cycle arrest, while also contributing to RAGE overexpression in fibroblasts. This activates signaling pathways such as ERK1/2, MAPK, and NF-κB, promoting pro-inflammatory cytokine secretion (e.g., TNF-α and IL-8). RAGE activation enhances extracellular matrix (ECM) production, including collagen types I and III, and stimulates matrix metalloproteinases (MMPs), impacting tissue remodeling and fibrosis. Created with BioRender.com (accessed on 14 November 2024).

The RAGE pathway plays a well-recognized role in diabetic wound development, though certain mechanisms remain incompletely understood. The accumulation of AGEs disrupts collagen fibril formation, leading to decreased scar elasticity, increased tissue contraction, and delayed wound closure [94]. Furthermore, the AGEs-induced expression of MMP9 has been identified as a key pathogenic factor hindering wound healing in diabetes [95,96]. Glycosylated ECM components contribute to fibroblast apoptosis and cause cell cycle arrest, further complicating the healing process [97]. Additionally, 3-deoxyglucosone, an upregulated precursor of AGEs in diabetic wounds, promotes fibroblast apoptosis via a RAGE-independent pathway involving NAD(P)H oxidase 4 (Nox4) [98]. Figure 4 illustrates the pathogenic effects of AGEs accumulation in diabetic wound formation.

AGEs also influence ECM turnover by upregulating RAGE expression, enhancing both collagen production and MMP2 activity. The stimulation of the AGEs/RAGE axis in fibroblasts activates ERK1/2 and NF-κB signaling, leading to the secretion of pro-inflammatory cytokines such as TNFα and IL-8. However, these effects are moderated by TGF-β, which stimulates the production of GM-CSF and IL-6 and, in synergy with RAGE, modulates anti-inflammatory cytokines IL-2 and IL-4. This dynamic interplay between RAGE and TGF-β is critical for balancing the pro- and anti-inflammatory processes essential for wound healing and fibrosis [99].

RAGE activation by other ligands similarly enhances the production of collagen types I and III and activates MMP1a and MMP9, contributing to ECM turnover [100]. Interestingly, studies indicate that administering exogenous RAGE ligands may promote angiogenesis and accelerate wound healing. These ligands compete with endogenous molecules for binding to RAGE, TLR2, and TLR4 receptors, thereby inhibiting NF-κB activation, reducing inflammation, and fostering tissue repair [101].

While the impact of the AGEs/RAGE pathway on diabetic wound formation is well established, its precise mechanisms warrant further investigation. AGEs accumulation and their precursors appear to impair wound healing directly, whereas RAGE activation involves complex interactions with other pathways, particularly the TGF-β axis. Nonetheless, limiting RAGE overexpression in diabetic wounds holds therapeutic potential by reducing excessive tissue damage and promoting effective healing [102].

### 4.8. Skin Cancer Progression

The RAGE pathway is widely believed to play a significant role in promoting tumor growth and metastasis across various cancers, particularly those of pulmonary, breast, and prostate origin [103,104,105]. This is partly due to its involvement in chronic inflammation, which fosters a pro-inflammatory tumor microenvironment, increases cytokine release, and helps tumors to evade immune surveillance [106]. Notably, studies have demonstrated that RAGE-deficient mice are resistant to induced skin carcinogenesis and unable to sustain inflammation, further underscoring the pathway’s role in cancer progression [107].

In the cutaneous environment, which is constantly exposed to carcinogenic UV radiation, the HMGB1/RAGE/ERK1/2 pathway has been shown to promote autophagy while inhibiting apoptosis in keratinocytes [108]. Additionally, the overexpression of the S100A8/A9 complex in squamous cell carcinoma activates the RAGE/ERK1/2 axis, driving keratinocyte proliferation and migration [109]. Recent studies suggest that HMGB1 and RAGE are also involved in the development of basal cell carcinoma and melanoma, although their roles differ across these skin cancer types [110,111,112].

Abe et al. demonstrated that AGEs, particularly those derived from glyceraldehyde and glycolaldehyde, promote the growth and migration of human melanoma cells [113]. Furthermore, HMGB1 and S100A9 have been identified as key RAGE and TLR4 ligands within the melanoma microenvironment. These ligands drive the conversion of monocytes into myeloid-derived suppressor cells (MDSCs), which inhibit the anti-tumor T cell response [114,115]. The S100A8/A9 complex also promotes melanoma progression through MCAM-dependent NF-κB activation and ROS production, a mechanism that may surpass the significance of the RAGE-dependent pathway [116].

Additionally, UV radiation exposure increases HMGB1 secretion, which, through RAGE, activates NF-κB and IRF3-dependent transcription of PD-L1 in melanocytes. This mechanism reduces T cell cytotoxicity and prevents melanocyte apoptosis, further facilitating tumor progression [117,118].

Another RAGE ligand, S100A4, is highly overexpressed in the melanoma microenvironment [119]. Tumor cells actively secrete S100A4 via an endoplasmic reticulum–Golgi-dependent mechanism, enhancing melanoma’s metastatic potential [120]. The paracrine secretion of S100A4 is believed to induce RAGE-dependent endothelial dysfunction, facilitating melanoma cell migration [121]. Other S100 family proteins are also implicated in melanoma progression, though their roles vary. For instance, S100P and S100B have been associated with promoting metastasis, while S100A2 is underexpressed in certain cases, suggesting a more complex, context-dependent function in melanoma [122,123,124].

Moreover, the lower levels of sRAGE and esRAGE have been identified as prognostic markers associated with reduced survival in melanoma patients [125]. These findings highlight the intricate roles of the RAGE pathway and its ligands in melanoma progression, providing insights into potential therapeutic targets for skin cancers.

### 4.9. Skin Aging

Throughout life, metabolites such as AGEs accumulate in the body, which has prompted growing interest in strategies to inhibit AGEs aggregation as they are believed to contribute to the aging process [126]. AGEs have been shown to accumulate in dermal elastin and collagen, where they interact with dermal fibroblasts, stimulating RAGE- and TGF-β-dependent fibrotic changes that promote skin aging [127]. Additionally, the glycation of the ECM appears to influence monocyte lineage differentiation, favoring the development of dendritic and macrophage-like cells, which contribute to a microinflammatory environment [128].

Studies in murine models have observed that dermal levels of AGEs, prostaglandin E2 (PGE2), TNF-α, and RAGE increase with age [129]. The RAGE pathway has also been implicated in melanogenesis and skin photoaging processes [130]. AGEs accumulation appears to make skin cells more susceptible to UV radiation by promoting the formation of reactive oxygen species (ROS) [131]. Furthermore, dysregulated transfer RNA-derived small RNAs (tsRNAs) linked to the AGE/RAGE pathway may contribute to UV-induced skin photoaging [132].

Overall, current research highlights the detrimental effects of AGEs on skin health and underscores the role of the RAGE pathway in skin photoaging. Targeting the RAGE pathway may provide promising approaches for anti-aging treatments, particularly through topical therapies in dermatology and cosmetology.

### 4.10. Other Cutaneous Conditions

The RAGE pathway may also be involved in several other dermatological conditions, though the current literature on these pathologies remains limited, and further studies are needed to confirm its role. Elevated serum levels of HMGB1 and moesin in severe acne patients suggest that the RAGE axis could contribute to acne pathogenesis [133]. Some acne therapies may exert their effects by modulating TNFα and AGE/RAGE signaling pathways [134].

Additionally, HMGB1 has emerged as a novel marker for systemic lupus erythematosus (SLE), with elevated levels detected both in the serum and skin lesions of SLE patients [135,136]. Increased RAGE expression in the microvascular endothelium, fibroblasts, and inflammatory cells has also been observed in paraffin-embedded tissue samples from patients with acquired reactive perforating collagenosis [137].

Elevated serum levels of S100A12, along with a subsequent decrease following treatment, suggest the potential involvement of the RAGE pathway in the pathogenesis of Behçet’s and Kawasaki’s diseases [138]. Notably, RAGE’s roles in angiogenesis and inflammation offer potential therapeutic applications, particularly in improving the survival of pedicled flaps and skin grafts in plastic surgery [139,140].

## 5. RAGE as a Treatment Target

**Figure 5:**
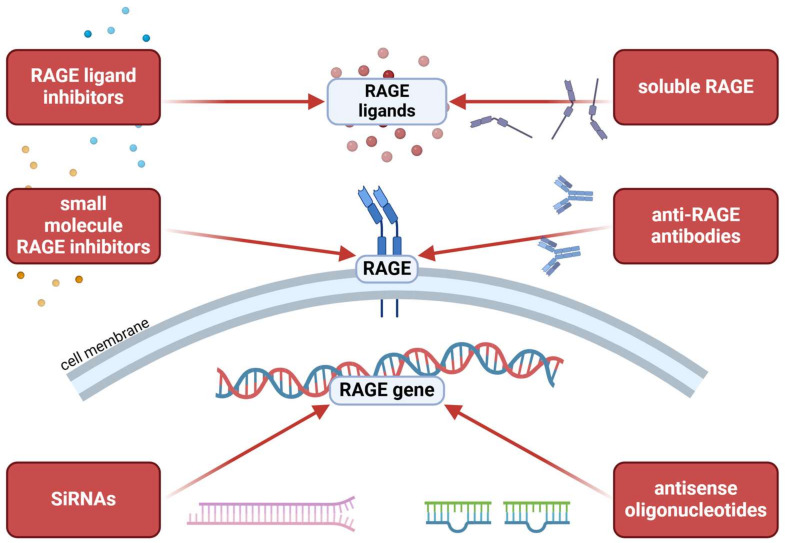
The RAGE pathway can be inhibited at three key intervention points: blocking RAGE ligands, inhibiting the receptor itself, and silencing the RAGE gene. Blue boxes highlight these intervention points, while red boxes illustrate various potential inhibitors of the RAGE pathway. Additional therapies can indirectly suppress contributing signaling pathways, providing complementary strategies for RAGE pathway inhibition. Created with BioRender.com (accessed on 14 November 2024).

Since RAGE is a multiligand receptor involved in numerous signaling pathways, its activity can be modulated at various stages of its signaling cascade. Figure 5 illustrates several potential strategies for targeting the RAGE pathway. In addition to therapies specifically targeting RAGE, various drugs are known to downregulate RAGE expression as part of their broader effects.

Among RAGE-specific treatments, small molecule inhibitors such as FPS-ZM1 and TTP488 (Azeliragon) directly block the ligand-binding domain of RAGE, preventing activation by pro-inflammatory ligands [141,142]. Monoclonal antibodies targeting RAGE represent another promising strategy, as they specifically bind to the receptor and obstruct ligand interactions, significantly reducing RAGE-mediated signaling [143,144]. Another approach involves sRAGE, a naturally occurring decoy receptor that sequesters RAGE ligands in circulation, thereby decreasing their availability to interact with membrane-bound RAGE [145].

Further strategies focus on inhibiting RAGE ligands, targeting specific molecules such as AGEs, HMGB1, and S100 proteins to reduce their interaction with RAGE and subsequent receptor activation [146,147]. Gene-silencing techniques, including small interfering RNA (siRNA) and antisense oligonucleotides, have also demonstrated efficacy in reducing RAGE expression at the transcriptional level, decreasing receptor availability on cell surfaces [148].

The RAGE pathway can also be downregulated indirectly by non-specific RAGE inhibitors, such as natural compounds with anti-RAGE properties (e.g., polyphenols and flavonoids) and medications including metformin, statins, angiotensin-converting enzyme (ACE) inhibitors, and angiotensin II receptor blockers (sartans) [149,150,151,152,153]. Additionally, anti-inflammatory agents targeting the NF-κB pathway and reactive oxygen species (ROS) inhibitors can modulate the RAGE signaling cascade, mitigating downstream inflammatory effects [154].

Given the pleiotropic effects of RAGE activation, RAGE inhibitors may influence a wide range of cell types and biological processes. While some clinical trials have demonstrated the safety of RAGE-specific inhibitors in non-dermatological diseases [155,156], these trials remain limited in number. Further data are needed to confirm their safety and efficacy for cutaneous applications. Topical formulations could offer a more targeted approach, potentially minimizing systemic side effects; however, additional clinical investigations are necessary to fully explore their potential in dermatological conditions.

### 5.1. RAGE-Modulating Therapies in Dermatology

While RAGE-targeting treatments with anti-inflammatory properties are not a novel concept in cutaneous pathologies, studies examining the effects of RAGE axis inhibition in dermatology remain limited and have primarily focused on non-specific RAGE modulators [157]. Table 5 summarizes the RAGE-targeting therapies studied to date for inflammatory dermatological conditions.

Network pharmacology has demonstrated that rutin and various active compounds found in Traditional Chinese Medicine (TCM) can downregulate the RAGE pathway. In murine models of imiquimod-induced psoriasis, these compounds have been shown to reduce inflammatory markers and improve skin lesions [158,159,160]. Additionally, Liu et al. reported that lipoxin A4 and its analog BML-111 may alleviate psoriatic dermatitis by inhibiting HMGB1 production, subsequently reducing the levels of RAGE, TLR4, and NF-κB [161].

In the context of atopic dermatitis (AD), several agents have demonstrated anti-inflammatory effects by modulating the RAGE pathway, particularly through the HMGB1 ligand. In a mouse model, a two-week daily administration of resveratrol or quercetin significantly decreased the expression of HMGB1, RAGE, NF-κB, PI3K, ERK1/2, TNFα, and IL-1β [162,163]. Similarly, reduced inflammatory infiltration was observed in mice treated with glycyrrhizin, tannic acid, and Coffea arabica extract, all of which influence the HMGB1/RAGE/NF-κB pathway [164,165,166].

Furthermore, network pharmacology and molecular docking studies suggest that TCM bioactive compounds, including Tripterygium wilfordii and Huangbai liniment, may alleviate lichen planus (LP) symptoms by inhibiting the AGE/RAGE/NF-κB pathway [167,168]. Quercetin, a primary component of Huangbai liniment, has demonstrated anti-inflammatory effects in LP by acting through the HMGB1/RAGE/NF-κB pathway. In vitro studies on activated T cells indicated that quercetin concentrations above 40 μM induced significant apoptosis, reduced IL-6 levels, and increased IFN-γ expression, reinforcing its potential therapeutic applications [169].

**Table 5:** Summary of RAGE-affecting therapies studied to date for inflammatory dermatological conditions (↑—increase, ↓—decrease).

Condition	Model	Medication	Type of Medication	Outcome	Year	Authors [Ref.]
Psoriasis	HSFs, HaCaTs	Rutin	Non-specific RAGE modulator	↓proliferation, ↓TNFα, ↓IL-6	2023	Wang et al. [158]
Psoriasis	NHEKs	Lipoxin A4	Non-specific RAGE modulator	↓HMGB1, ↓RAGE, ↓TLR4, ↓ERK1/2	2017	Liu et al. [161]
Psoriasis	Murine	Shenling Baizhu powder	Non-specific RAGE modulator	↓PASI, ↓skin thickness, ↓proliferation, ↓IL-17	2024	Tang et al. [160]
Psoriasis	Murine	Xijiao Dihuang Decoction	Non-specific RAGE modulator	↓PASI, ↓skin thickness, ↓angiogenesis, ↓MMP9, ↓STAS3, ↓VEGFA, ↓TNFα, ↓IL-6	2023	Guo et al. [159]
Psoriasis	Murine	Rutin	Non-specific RAGE modulator	↓PASI, ↓skin thickness, ↓inflammatory cells infiltration, ↓NFκB, ↓RAGE	2023	Wang et al. [158]
Psoriasis	Murine	BML-111	Non-specific RAGE modulator	↓PASI, ↓erythema, ↓skin thickness, ↓HMGB1, ↓RAGE, ↓TLR4, ↓ERK1/2, ↓NF-κB, ↓IL-1β, ↓TNFα, ↓IL-6, ↓IL-17a, ↓IL-17c, ↓IL-23, ↓IL-22	2017	Liu et al. [161]
Atopic dermatitis	HaCaTs	Coffea arabica	Non-specific RAGE modulator	↓ROS, ↓ERK1/2, ↓p38, ↓NFκB, ↓NLRP3, ↓TNFα, ↓IL-6, ↓HMGB1, ↓RAGE, ↑filaggrin, ↑claudin-1	2023	Chang et al. [166]
Atopic dermatitis	Mouse mastocytoma cell line	Glycyrrhizin	Non-specific RAGE modulator	↓ERK1/2, ↓PI3K, ↓RAGE, ↓NFκB, ↓TNFα, ↓IL-6, ↓pAKT, ↓MC tryptase	2018	Wang et al. [164]
Atopic dermatitis	Murine	Coffea arabica	Non-specific RAGE modulator	↓skin thickness, ↓erythema, ↓TNFα, ↓TSLP	2023	Chang et al. [166]
Atopic dermatitis	Murine	Glycyrrhizin	Non-specific RAGE modulator	↓IgE, ↓dermatitis, ↓mast cells, ↓HMGB1, ↓RAGE, ↓NFκB, ↓TNFα, ↓IL-6	2018	Wang et al. [164]
Atopic dermatitis	Murine	Tannic acid	Non-specific RAGE modulator	↓cell proliferation, ↓skin thickness, ↓neutrophil and mast cells infiltration, ↑PPARγ, ↓TNFα, ↓HMGB1, ↓RAGE, ↓ERK1/2, ↓NFκB, ↓COX2, ↓IL-1β, ↓IFNγ, ↓IL-4	2015	Karuppagounder et al. [165]
Atopic dermatitis	Murine	Quercetin	Non-specific RAGE modulator	↓skin thickness, ↓inflammatory cells infiltration, ↑Nrf2, ↓HMGB1, ↓RAGE, ↓NFκB, ↓ERK1/2, ↓COX2, ↓TNFα, ↓IL-1β, ↓IL-2Rα, ↓IFNγ, ↓IL-4	2015	Karuppagounder et al. [163]
Atopic dermatitis	Murine	Resveratrol	Non-specific RAGE modulator	↓skin thickness, ↓inflammatory and mast cells infiltration, ↓GRP78, ↓CHOP, ↓cleaved caspase-7, ↓HMGB1, ↓RAGE, ↓NFκB, ↓PI3K, ↓ERK1/2, ↓COX2, ↓TNFα, ↓IL-1β, ↓IL-2Rα, ↓IFNγ, ↓IL-4	2014	Karuppagounder et al. [162]
Lichen planus	CD3+ T lymphocytes	Quercetin	Non-specific RAGE modulator	↓proliferation, ↓migration, ↑apoptosis, ↑IFN-γ, ↓IL-6	2022	Zhao et al. [169]

Abbreviations: 5(S)-6(R)-7-trihydroxyheptanoic acid methyl ester (BML-111), C/EBP Homologous Protein (CHOP), Extracellular Signal-Regulated Kinases 1 and 2 (ERK1/2), Glucose-Regulated Protein 78 (GRP78), High Mobility Group Box 1 (HMGB1), Human Adult Keratinocytes (HaCaTs), Human Skin Fibroblast (HSFs), immunoglobulin E (IgE), Interferon gamma (IFNγ), Interleukin (IL), Interleukin-2 Receptor alpha (IL-2Rα), Matrix Metalloproteinase-9 (MMP9), Nuclear Factor kappa-light-chain-enhancer of activated B cells (NFκB), Nod-like receptor protein 3 (NLRP3), Nuclear factor erythroid 2–related factor 2 (Nrf2), Peroxisome Cyclooxygenase-2 (COX2), Phosphoinositide 3-Kinase (PI3K), Proliferator-Activated Receptor gamma (PPARγ), Psoriasis Area and Severity Index (PASI), Receptor for advanced glycation end-products (RAGE), reactive oxygen species (ROS), Suppressor of Tumorigenicity 3 (STAS3), Thymic Stromal Lymphopoietin (TSLP), Toll-like receptor 4 (TLR4), tumor necrosis factor alpha (TNFα), Vascular Endothelial Growth Factor A (VEGFA), phosphorylated Protein Kinase B (pAKT).

### 5.2. Targeting RAGE to Improve Wound Healing

The most extensively studied indication for cutaneous RAGE inhibition is the enhancement of diabetic wound healing [102]. Table 6 provides an overview of anti-RAGE medications currently under investigation for their effectiveness in wound healing contexts.

Studies have shown that RAGE inhibition, achieved through blocking antibodies or the administration of sRAGE, significantly enhances diabetic wound healing in animal models [170,171,172]. Recognizing the short half-life of sRAGE in the proteolytic environment of wounds, Kang et al. developed a recombinant fusion protein comprising the binding domain of RAGE (vRAGE) linked to elastin-like polypeptides (ELPs), offering improved stability and efficacy [173]. Additionally, topical applications of sRAGE, antioxidants, and gold nanoparticles have demonstrated promising results in promoting healing in diabetic wounds, underscoring the accessibility of skin diseases to localized treatments [174,175,176,177].

Recent studies suggest that several plant-derived substances and active compounds from Traditional Chinese Medicine (TCM) may enhance wound healing by downregulating RAGE pathway activity [178,179,180,181,182]. Other compounds, including resveratrol, aminoguanidine, Centella cordifolia, ibrutinib, and N-acetyl-L-cysteine, have also been reported to improve wound healing by modulating RAGE expression [183,184,185,186,187]. Furthermore, small extracellular vesicles derived from human decidua-derived mesenchymal stem cells (dMSC-sEVs) and miRNA-221-3p found in endothelial progenitor cell-derived exosomes have demonstrated efficacy in inhibiting RAGE activity and enhancing dermal conditions in diabetic mice [188,189]. Notably, RAGE axis inhibition has also shown promise in improving wound healing in non-diabetic contexts [190].

Conversely, Ranzato et al. observed in cellular models that activating RAGE with HMGB1 may enhance wound healing capacity [191,192]. Subsequent murine studies have further reported that HMGB1 can promote wound healing through the RAGE pathway [193]. These findings highlight the complex role of the RAGE pathway in skin wound healing, suggesting that its effects may vary based on wound etiology. Tissue damage caused by diabetes differs significantly from that resulting from mechanical injuries or burns, which may explain the differing outcomes. Additionally, the impact of the RAGE pathway may depend on the specific ligands involved, as certain ligands can engage other receptors beyond RAGE, influencing multiple signaling pathways.

**Table 6:** A summary of anti-RAGE therapies currently being explored for their potential applications in wound healing (↑—increase, ↓—decrease).

Condition	Model	Medication	Type of Medication	Outcome	Year	Authors [Ref.]
Non-diabetic wound	HSFs	THC/HGF	Non-specific RAGE modulator	↓HMGB1, ↓RAGE, ↓pNF-κB, ↑BCL-2, ↓BAX, ↓cleaved caspase-3, ↓TNFα, ↓IL-6, ↓TGF-β, ↓α-SMA, ↑COL3A1, ↓FN1, ↓COL1A1	2024	Xing et al. [176]
Diabetic wound	HSFs	Dang-Gui-Si-Ni decoction	Non-specific RAGE modulator	↓α-SMA, ↓collagen I, ↓Smad2, ↓AGEs, ↓RAGE, ↑TGF-β1, ↑Smad3	2024	Zhang et al. [181]
Diabetic wound	HSFs	dMSC-sEVs	Non-specific RAGE modulator	↑proliferation, ↑migration, ↑α-SMA, ↑collagen I, ↑Smad, ↓RAGE	2020	Bian et al. [188]
Diabetic wound	HaCaTs	N-acetyl-L-cysteine	Non-specific RAGE modulator	↓AGEs, ↑cell viability, ↑cell migration ↓IL-6, ↓IL-8, ↓MMP9, ↓NF-κB,	2017	Yang et al. [187]
Diabetic wound	Porcine	Anti-RAGE antibody	RAGE-specific inhibitor	↓wound size, ↑collagen, ↓RAGE, ↓Mac, ↓IL-6	2022	Johnson et al. [170]
Non-diabetic wound	Murine	THC/HGF	Non-specific RAGE modulator	↓fibrosis, ↑regular collagen fibers, ↑epidermis thickness, ↑angiogenesis, ↑CD31, ↑CD206, ↓INOS, ↓HMGB1, ↓RAGE, ↓TGF-β	2024	Xing et al. [176]
Diabetic wound	Murine	Dang-Gui-Si-Ni decoction	Non-specific RAGE modulator	↓wound size, ↓IL-1β, ↓IL-6, ↓TNFα, ↓AGEs, ↓RAGE, ↑INS, ↑Ang-1, ↑VEGF, ↑Tie-2, ↑TGF-β1, ↑Smad3, ↓Smad2	2024	Zhang et al. [181]
Non-diabetic wound	Murine	Cucurbitaceae seed oils	Non-specific RAGE modulator	↓wound size, ↑epidermis thickness, ↓AGEs, ↓RAGE, ↑Nrf2, ↑HO-1, ↓TNF-α, ↓NF-κB, ↓NLRP3, ↓CX-43, ↓EGF	2024	Emad et al. [182]
Diabetic wound	Murine	Resveratrol	Non-specific RAGE modulator	↓wound size, ↑epidermis thickness, ↓IL-1β, ↓IL-6, ↓IL-18, ↓TNF-α, ↓RAGE, ↓NF-κB	2023	Youjun et al. [183]
Diabetic wound	Murine	vRAGE-ELP/SDF1-ELP	Decoy receptor/angiogenic chemokine	↓wound size, ↑skin thickness, ↑CD31	2023	Kang et al. [177]
Diabetic wound	Murine	Polygonatum kingianum (polygonati rhizome)	Non-specific RAGE modulator	↓wound size, ↓inflammatory infiltration, ↑angiogenesis, ↑skin thickness, ↓AGEs, ↓RAGE, ↑Nrf2, ↑HO-1, ↑CD34, ↑bFGF, ↑VEGF, ↓SOD, ↓GSH, ↓MMP-9, ↓MMP-2, ↑TIMP-2, ↓GSP, ↓GHb, ↓ICAM-1, ↑T-AOC, ↑SOD, ↑FINS, ↓MDA, ↓TNFα, ↓IL-6, ↓IL-2, ↓IFN-γ	2022	Pan-Yue et al. [180]
Diabetic wound	Murine	vRAGE-ELP	Decoy receptor	↓wound size, ↓epithelial gap	2021	Kang et al. [173]
Diabetic wound	Murine	dMSC-sEVs	Non-specific RAGE modulator	↓wound size, ↑collagen, ↑PCNA, ↑CXCR4, ↑α-SMA, ↓p21	2020	Bian et al. [188]
Diabetic and non-diabetic wound	Murine	miRNA-221-3p	Non-specific RAGE modulator	↓wound size, ↑VEGF, ↑CD31, ↑Ki67	2020	Xu et al. [189]
Diabetic wound	Murine	Ibrutinib	Bruton tyrosine kinase inhibitor	↓wound size, ↓IL-1β, ↓TNF-α, ↓IL-6, ↓TLR2, ↓TLR4, ↓RAGE, ↓NF-κB	2019	Yang et al. [186]
Diabetic wound	Murine	sRAGE/SDF-1	Decoy receptor/angiogenic chemokine	↓wound size	2016	Olekson et al. [172]
Burn wound	Murine	Thymosin beta 4	Non-specific RAGE modulator	↓wound size, ↑granulation, ↑Ki67, ↑angiogenesis, ↓TNFα, ↓IL-1β, ↑VEGF, ↓RAGE	2014	Kim et al. [190]
Diabetic wound	Murine	Aminoguanidine	Non-specific RAGE modulator	↓inflammatory infiltrate, ↓AGEs, ↓RAGE, ↓NF-κB	2013	Tian et al. [184]
Diabetic wound	Murine	AuNP/EGCG/ALA	Non-specific RAGE modulator	↓wound size, ↓RAGE, ↑VEGF, ↓monocyte infiltration	2012	Chen et al. [175]
Diabetic wound	Murine	sRAGE	Decoy receptor	↑neovascularization, ↑granulation, ↓epithelial gap	2004	Wear-Maggitti et al. [174]
Diabetic wound	Murine	sRAGE	Decoy receptor	↓wound size, ↓TNFα, ↓IL-6, ↓MMP2, ↓MMP3, ↓MMP9, ↑PDGF-B, ↑VEGF	2001	Goova et al. [171]

Abbreviations: advanced glycation end-products (AGEs), Alpha-Smooth Muscle Actin (α-SMA), Angiopoietin 1 (Ang-1), B-Cell Lymphoma 2 protein (BCL-2), BCL-2-Associated X Protein (BAX), Binding domain of RAGE linked to elastin-like polypeptides (vRAGE-ELPs), C-X-C Chemokine Receptor Type 4 (CXCR4), Collagen Type I Alpha 1 Chain (COL1A1), Collagen Type III Alpha 1 Chain (COL3A1), Cluster of Differentiation (CD), Epigallocatechin gallate (EGCG), Fibronectin 1 (FN1), Fibroblast Growth Factor (FGF), Fasting Insulin (FINS), Glutathione (GSH), Glycated Serum Protein (GSP), Glycosylated Hemoglobin (GHb), High Mobility Group Box 1 (HMGB1), Heme Oxygenase 1 (HO-1), Human Adult Keratinocytes (HaCaTs), Human Skin Fibroblast (HSFs), Inducible Nitric Oxide Synthase (iNOS), Insulin (INS), Intercellular Adhesion Molecule 1 (ICAM-1), Interleukin (IL), Matrix Metalloproteinase (MMP), Mothers Against Decapentaplegic Homolog (Smad), Malondialdehyde (MDA), Nuclear Factor Erythroid 2–Related Factor 2 (Nrf2), Phosphorylated Nuclear Factor Kappa B (pNF-κB), Platelet-Derived Growth Factor Subunit B (PDGF-B), Proliferating Cell Nuclear Antigen (PCNA), Receptor for advanced glycation end-products (RAGE), small extracellular vesicles derived from human decidua-derived mesenchymal stem cells (dMSC-sEVs), Stromal Cell-Derived Growth Factor-1 Alpha-Elastin-Like Peptide (SDF1-ELP), Superoxide Dismutase (SOD), soluble receptor for advanced glycation end-products (sRAGE), tetrahydrocurcumin and hepatocyte growth factor (THC/HGF), Total Antioxidant Capacity (T-AOC), Toll-Like Receptor (TLR), Transforming Growth Factor Beta (TGF-β), tyrosine kinase with immunoglobulin-like and EGF-like domains 2 (Tie-2), tumor necrosis factor alpha (TNFα), Vascular Endothelial Growth Factor (VEGF).

### 5.3. Anti-RAGE Therapy in Skin Cancer

In a murine lung cancer model, the administration of sRAGE significantly reduced both the growth and metastasis of implanted and spontaneously developing tumors [194]. This effect is believed to stem from the RAGE-dependent inhibition of critical signaling pathways, including p44/p42, p38, and SAP/JNK MAP kinases, which play essential roles in tumor proliferation, invasion, and MMP expression.

These findings have been extended to skin cancer research, where RAGE-neutralizing antibodies have demonstrated efficacy in inhibiting tumor formation in melanoma xenografts within athymic mice. In tumor-bearing mice, anti-RAGE treatment not only improved survival rates but also reduced lung metastasis [113]. Additionally, treatment with sRAGE has been shown to enhance endothelial integrity, potentially decreasing metastasis [121]. Table 7 provides a summary of RAGE-modulating therapies investigated for skin cancer treatment.

Several active compounds from Traditional Chinese Medicine (TCM) with anti-cancer properties have also shown potential for reducing angiogenesis in cancer by modulating the AGE/RAGE and PD-1 pathways in cell culture models [195]. Notably, Matsushita et al. reported a case in which MK615, an extract from Japanese apricot containing natural compounds such as triterpenoids, suppressed the in-transit metastasis of malignant melanoma and increased the tumor apoptotic index [196]. In further cell culture analyses, MK615 inhibited RAGE expression and suppressed HMGB1 release in melanoma cells.

**Table 7:** Summary of RAGE-modulating therapies investigated for skin cancer treatment (↑—increase, ↓—decrease).

Condition	Model	Medication	Type of Medication	Outcome	Year	Authors [Ref.]
Melanoma	Human melanoma cell line A375	sRAGE	Decoy receptor	↓cell migration	2016	Herwig et al. [120]
Melanoma	Human melanoma cell line SK-MEL28	MK615	Non-specific RAGE modulator	↓proliferation, ↑apoptosis, ↓RAGE, ↓HMGB1	2010	Matsushita et al. [196]
Melanoma	Human melanoma cell line G361 and A375	Anti-RAGE antibody	RAGE-specific inhibitor	↓proliferation	2004	Abe et al. [113]
Melanoma	Murine	Anti-RAGE antibody	RAGE-specific inhibitor	↓tumor size, ↓lung metastasis, ↑mice surivial	2004	Abe et al. [113]
Tumor angiogenesis	HUVECs	Centella asiatica	Non-specific RAGE modulator	↓proliferation, ↓cell migration, ↓vascular tube formation	2013	Zhu et al. [122]

Abbreviations: extract from Japanese apricot (MK615), High Mobility Group Box 1 (HMGB1), Human Umbilical Vein Endothelial Cells (HUVECs), soluble receptor for advanced glycation end-products (sRAGE).

These findings suggest that anti-RAGE therapies hold promise for melanoma patients, although their efficacy in other skin cancers remains largely unexplored. Continued research is needed to evaluate the potential of RAGE-targeting therapies across diverse types of skin cancer and to explore their mechanisms of action further.

### 5.4. Targeting RAGE to Decelerate Skin Aging

While UV radiation is well-known to accelerate skin aging, recent studies suggest that certain compounds may decelerate this process by suppressing the RAGE pathway. For instance, Han et al. demonstrated that plantamajoside, derived from Plantago asiatica, exerts anti-inflammatory and antioxidative effects in cultured human keratinocytes and fibroblasts by downregulating RAGE and NF-κB expression [197]. Other compounds reported to alleviate skin photoaging and glycation through the inhibition of the AGE/RAGE pathway include gentiopicroside, Chenopodium formosanum, Pogostemon cablin (patchouli), and Ba Zhen Tang [198,199,200,201]. Table 8 summarizes proposed RAGE modulation therapies aimed at decelerating skin aging.

Additionally, Li et al. observed that carnosine may counteract skin aging by promoting the macrophage-mediated clearance of senescent keratinocytes and fibroblasts through CD36- and RAGE-dependent activation of the AKT2 pathway [202]. Rodent studies further support that substances downregulating RAGE expression can reduce skin aging and photodamage [203].

**Table 8:** A summary of proposed RAGE modulation therapies for decelerating skin aging (↑—increase, ↓—decrease).

Condition	Model	Medication	Type of Medication	Outcome	Year	Authors [Ref.]
AGEs exposure, UVB irradiation	HaCaTs	Plantamajoside	Non-specific RAGE modulator	↑cell viability, ↓ROS, ↓RAGE, ↓MMP1, ↓TNFα, ↓IL-1β, ↓NF-ĸB/p65	2016	Han et al. [197]
AGEs exposure, UVB irradiation	HSFs	Plantamajoside	Non-specific RAGE modulator	↓RAGE, ↓MMP1	2016	Han et al. [197]
AGEs exposure, UVB irradiation	HSFs	Chenopodium formosanum	Non-specific RAGE modulator	↓ROS, ↑Nrf2, ↑HO-1, ↓MMP1, ↓MMP3, ↓MMP9, ↑TIMP-1, ↓MAPK, ↓AP-1, ↓RAGE, ↑collagen	2022	Lyu et al. [199]
UVB irradiation	HaCaTs	Ba Zhen Tang	Non-specific RAGE modulator	↓SA-β-gal, ↓p16INK4a	2022	Han et al. [201]
UVB irradiation	Murine	Schizonepeta tenuifolia	Non-specific RAGE modulator	↓skin wrinkles, ↓skin thickness, ↑collagen, ↓MMPs, ↑TIMP-1, ↓skin dehydration, ↑hyaluronic acid, ↓MAPK, ↓NF-κB, ↓AGEs ↓RAGE	2023	Gu et al. [203]
MGO exposure	CCC-ESF-1	Gentiopicroside	Non-specific RAGE modulator	↓CML, ↑cell viability, ↑FN-1, ↑LM-5, ↑COL-1, ↓MMP2, ↓MMP9, ↓ROS, ↓IL-6, ↓IL-8, ↓IL-1β, ↓NF-κB, ↓RAGE	2024	Chen et al. [198]

Abbreviations: Activator Protein 1 (AP-1), advanced glycation end-products (AGEs), Collagen Type 1 (COL-1), Cyclin-Dependent Kinase Inhibitor 2A (p16INK4a), Fibronectin 1 (FN-1), Heme Oxygenase-1 (HO-1), Human Adult Keratinocytes (HaCaTs), Human Embryonic Skin Fibroblast (CCC-ESF-1), Human Skin Fibroblasts (HSFs), Interleukin (IL), Laminin 5 (LM-5), Matrix Metalloproteinase (MMP), Mitogen-Activated Protein Kinase (MAPK), Nε-Carboxymethyllysine (CML), Nuclear Factor Erythroid 2-Related Factor 2 (Nrf2), Nuclear Factor Kappa-Light-Chain-Enhancer of Activated B Cells (NF-κB), receptor for advanced glycation end-products (RAGE), reactive oxygen species (ROS), Senescence-Associated β-galactosidase (SA-β-gal), Tissue Inhibitor of Metalloproteinases 1 (TIMP-1), tumor necrosis factor alpha (TNFα), Ultraviolet B (UVB).

However, most of these compounds act as non-specific RAGE inhibitors, impacting multiple pathways. RAGE-specific inhibitors have yet to be extensively studied for their effects on skin aging, underscoring the need for further research into their targeted applications in dermatology.

## 6. Conclusions

In conclusion, the RAGE pathway plays an integral role in skin homeostasis and is implicated in a variety of dermatological conditions, particularly those associated with immune dysregulation. The accumulation of AGEs and other RAGE ligands is central to the pathogenesis of numerous skin diseases, highlighting RAGE as a promising therapeutic target. Soluble forms of RAGE show potential both as therapeutic agents and as biomarkers for tracking disease progression; however, the role of dnRAGE in skin disorders remains largely unexplored and warrants further investigation. While RAGE-targeted strategies have been established for enhancing wound healing in diabetic contexts, the efficacy of specific anti-RAGE therapies for other skin conditions has yet to be validated. Despite these advances, the field faces significant limitations. For instance, fl-RAGE expression in skin cells from inflammatory skin diseases such as psoriasis or atopic dermatitis has not been thoroughly studied, leaving a critical gap in understanding its direct involvement in these conditions. Furthermore, most existing studies do not adequately distinguish between the roles of distinct RAGE isoforms, which hinders the development of targeted and effective therapies. Experimental models that accurately mimic the complexity of human skin diseases are also lacking, posing a challenge for translating preclinical findings into clinical applications. Future research should focus on addressing these limitations by elucidating the specific contributions of different RAGE isoforms in various skin disorders and developing advanced models for studying RAGE-related mechanisms. Additionally, investigating the interplay between RAGE and other inflammatory pathways could provide valuable insights into the broader context of immune-mediated skin diseases. Overcoming these challenges will be essential for advancing the field and unlocking the full therapeutic potential of targeting the RAGE pathway.

## Figures and Tables

**Table 3:** Summary of current human-based studies on RAGE involvement in lichen planus (LP), emphasizing its potential role in disease pathogenesis (↑—increased concentration).

Material	Method	Study Size	Markers	Year	Authors [Ref.]
Serum	ELISA	50	↑S100A8/A9	2016	de Carvalho et al. [69]
Skin biopsy (dermis)	IHC	49	↑HMGB1, ↑RAGE	2018	de Carvalho et al. [70]
Mucosa biopsy	IHC	45	↑HMGB1, ↑RAGE	2018	Salem et al. [71]
Skin biopsy	IHC	50	↑S100A8	2016	de Carvalho et al. [69]
Skin biopsy	PCR	50	↑S100A8, ↑S100A9, ↑S100A8/A9	2016	de Carvalho et al. [69]

Abbreviations: Enzyme-Linked Immunosorbent Assay (ELISA), High Mobility Group Box 1 (HMGB1), immunohistochemistry (IHC), Polymerase Chain Reaction (PCR), receptor for advanced glycation end-products (RAGE).

## Supplementary Materials

The following supporting information can be downloaded at https://www.mdpi.com/article/10.3390/ijms252413570/s1.
