# Supplementary material for: The RAGE Pathway in Skin Pathology Development: A Comprehensive Review of Its Role and Therapeutic Potential
Source: Int J Mol Sci. 2024 Dec 18;25(24):13570. doi: 10.3390/ijms252413570 (PMC11676465; doi:10.3390/ijms252413570)
Supplement: Supplementary file 1 [file ijms-25-13570-s001.zip › ijms-3363947-supplementary.pdf]

SUPPLEMENTARY DATA

Supplementary Materials and Methods:

Literature search

The selected literature (n = 1191) was retrieved from PubMed (https://pubmed.ncbi.nlm.nih.gov/; accessed on 21 September 2024) without any time frame restrictions. Search terms included 'RAGE,' 'Skin,' 'Cutaneous,' 'Dermatology,' 'Disease,' 'Inflammation,' 'Fibrosis,' 'Wound,' 'Aging,' 'UV,' 'Keratinocytes,' and 'Fibroblasts,' combined in various configurations with the Boolean operators 'AND' and 'OR.'"

Literature screening

After the final selection, duplicate studies and articles published in languages other than English were removed. Articles with abstracts not related to the RAGE pathway were excluded, along with research papers discussing the RAGE axis in non-cutaneous conditions. This process left 180 papers for eligibility assessment. Articles unrelated to RAGE signaling or RAGE-modulating treatment in skin diseases were then excluded. Reviews, comments, and editorials were also omitted, resulting in 134 articles selected for final analysis. The literature review was conducted from September to November 2024, and the screening process is illustrated in **Supplementary Figure 1**.

Supplementary Figure

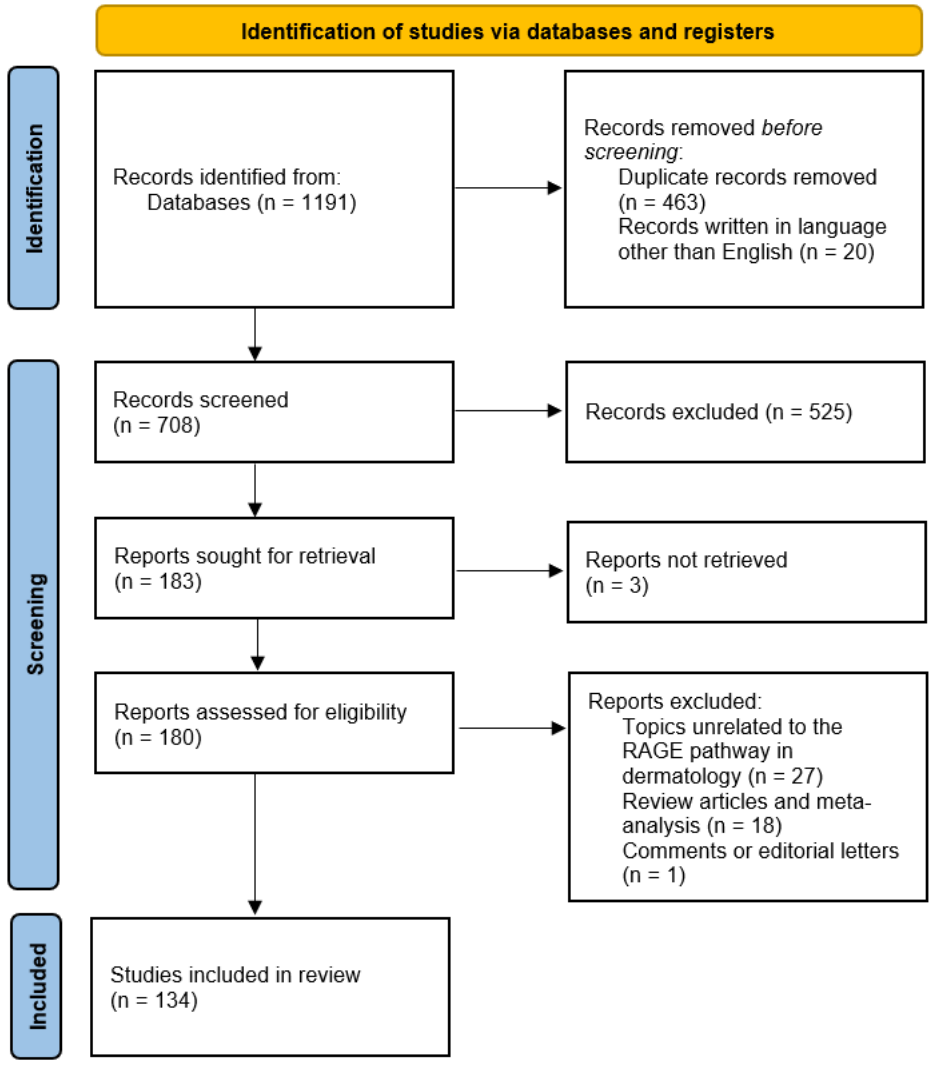

Supplementary Figure S1. Inclusion scheme for relevant publications.
